# Proteomic Insights Into Human Induced Regulatory T Cells: The Role of LPXN and CD160 in Immune Suppression

**DOI:** 10.1016/j.mcpro.2026.101644

**Published:** 2026-08-20

**Authors:** Kedar Batkulwar, Syed Bilal Ahmad Andrabi, Roosa Kattelus, Tanja Buchacher, Ilona Arnkil, Inna Starskaia, Omid Rasool, Robert Moulder, Riitta Lahesmaa

**Affiliations:** 1 Turku Bioscience Centre, University of Turku and Åbo Akademi University, Turku, Finland; 2 InFLAMES Research Flagship Center, University of Turku and Åbo Akademi University, Turku, Finland; 3 Institute of Biomedicine, University of Turku and Åbo Akademi University, Turku, Finland

**Keywords:** CD4, CD160, FOXP3, LPXN, mass-spectrometry, proteomics, regulatory T-cells, T-cells

## Abstract

Regulatory T cells (Treg) play a central role in maintaining immune homeostasis, and the implementation of *in vitro* induced Treg cells (iTreg) to control immune function has significant potential in clinical medicine. The clinical application of iTreg has been limited by their poor stability. To better define the molecular characteristics of human iTreg, we performed a data-independent acquisition proteomics, detecting over 8000 proteins and providing a quantitative comparison of their relative levels in iTreg and activated Th0 cells. Consistent with the known molecular characteristics of Treg, several Treg signature proteins, including FOXP3, IKZF4, IL2RA, CTLA4, PD-1, IKZF3, LAG3, RUNX1 and hypermethylated in cancer 1, were identified and validated using Tier two targeted SRM and/or qRT-PCR. Notably, Leupaxin level was upregulated during Treg cell differentiation. Functional studies demonstrated that LPXN-deficient cells showed impaired expression of Treg protein markers FOXP3, IKZF4 and IKZF3 and impaired suppression of effector T cells. In addition, we identified a distinct CD160^+^ iTreg subpopulation characterized by a distinct proteomic signature as compared to CD160^−^ iTreg. Together, these findings provide a high-resolution proteomic landscape of human iTreg and identified a novel role of LPXN in the development and suppressive activity of iTreg.

## Highlights

• A comprehensive protein landscape of human induced regulatory T cells (iTreg) was determined by data-independent acquisition mass spectrometry.
• Leupaxin-deficient iTreg exhibited impaired suppressive function and reduced expression of FOXP3 and the transcription factors IKZF3 and IKZF4.
• A CD160⁺ iTreg subpopulation with a distinct proteomic profile was identified.

## Graphical Abstract

**Figure:**
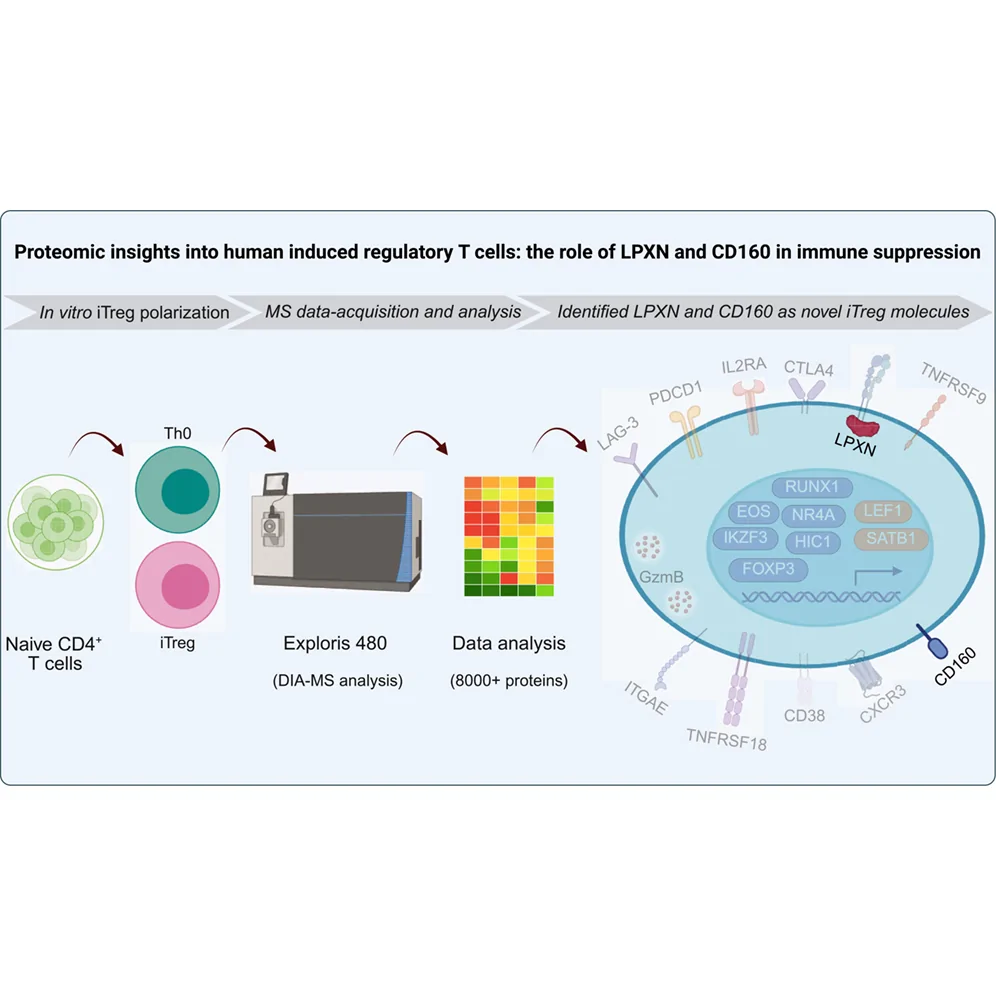

## In Brief

Using data-independent acquisition proteomics, we define the molecular landscape of human induced regulatory T cells, detecting over 8000 proteins and validating key Treg signature molecules. We uncover a role for leupaxin in iTreg differentiation and suppressive function and identify a distinct CD160⁺ iTreg subpopulation with a unique proteomic profile.

Regulatory T cells (Treg) are a subset of CD4^+^ T cells that can regulate and prevent excessive immune responses (1). Treg are typically produced in the thymus (tTreg), although some T cells can acquire the regulatory phenotype in the periphery and are referred to as peripheral Treg (pTreg). Treg modulate the immune response through inhibitory receptors (2, 3) and cytokines (4, 5, 6), and are essential for maintaining immune tolerance and preventing immune-mediated tissue damage. However, when their function is disrupted, inflammation and autoimmunity may occur (1, 7, 8). Understanding the mechanisms that preserve Treg integrity is therefore crucial, as any disruption may impair their suppressive function (9, 10, 11).

FOXP3 is a key transcription factor (TF) involved in the development and function of Treg (12, 13). In addition to FOXP3, several other proteins, including CD25, IKZFs, CTLA-4, GITR, NR4As, C-REL, NFAT, SMAD factors, STAT5, and RUNX1 regulate the function of Treg (14, 15, 16, 17, 18, 19). Earlier, we identified a novel role for the TF hypermethylated in cancer 1 (HIC1) in the differentiation of human iTreg (20). Consistent with these observations, other studies have shown that Treg can suppress effector T-cell expansion in a FOXP3-independent manner (21).

Mass spectrometry–based proteomics has been used to characterize Treg, uncovering key signaling pathways and molecules that are essential for their development and stability (22, 23, 24, 25, 26). These studies have also identified surface markers that define the functional heterogeneity of Treg and protein signatures that distinguish iTreg from conventional T cells (Tconv) and naïve Treg. With advances in technology, faster and more sensitive mass spectrometers together with improved data acquisition and analysis now allow more comprehensive protein profiling. Data-independent acquisition mass spectrometry (DIA-MS) is a powerful proteomics approach that allows the detection and quantification of a wide range of proteins simultaneously (27, 28). In this study, DIA-MS was used to generate a comprehensive iTreg proteome, shedding new light on their molecular features and functional characteristics. Notably, our findings highlight the role of Leupaxin in modulating the suppressive function of iTreg by regulating the expression of key Treg-associated proteins. Furthermore, CD160 was detected in a subpopulation of iTreg, and was found to be enriched exclusively in iTreg compared with other *in vitro*–induced CD4^+^ T-cell subsets.

## Experimental Procedures

### Experimental Design and Statistical Rationale

To characterize the proteomic signature of human iTreg cells, we performed DIA-MS profiling using three independent biological replicates to capture biologically relevant variability. Technical replicates were not included due to the high reproducibility of DIA-based workflows. Key findings were validated by Tier two targeted SRM assay in three more biological replicates, whilst other verifications were made using Western blotting and flow cytometry in additional biological replicates.

Proteomic data were processed and quantified using Spectronaut v18. Protein expression profiles were compared between iTreg and Th0 cells. Proteins with a log_2_ fold change greater than 1 and a false discovery rate (FDR) below 0.05, were considered significantly differentially expressed. GraphPad Prism v10 was used to generate a volcano plot illustrating upregulated and downregulated proteins. Principal component analysis (PCA) and heatmap visualization of differentially expressed proteins were performed in Perseus v1.6.2.3. Upstream regulator and pathway enrichment analyses were performed using Ingenuity Pathway Analysis (IPA, Qiagen) and Advaita’s iPathwayGuide platform.

### Primary Human CD4^+^ T-Cell Isolation and iTreg Cell Polarization

Human mononuclear cells were isolated from umbilical cord blood by density gradient centrifugation using Ficoll-Paque Plus (Cytiva, Cat# 17144003). CD4^+^ T cells were purified from mononuclear cells by positive selection using CD4^+^ Dynabeads (Invitrogen, Cat# 11331D), followed by depletion of CD25^+^ T cells with CD25 MicroBeads II (Miltenyi Biotec, Cat# 130-092-983), as previously described (26). Prior to activation, proportion of naïve CD4^+^CD25^−^ T cells from different donors were confirmed by flow cytometry to be CD45RA^+^ and CD45RO^−^.

The CD4^+^CD25^−^ cells were activated with plate-bound anti-CD3 (1000 ng/well, Beckman Coulter, Cat# IM1304) and soluble anti-CD28 (1000 ng/ml Beckman Coulter, Cat# IM1376) antibodies in X-vivo 15 serum-free medium (Lonza). To induce iTreg differentiation, cultures were supplemented with 12 ng/ml IL-2 (Cat# 202-IL-010), 10 ng/ml TGF-β (R&D Systems, Cat# 240-B), 10 nM all-trans retinoic acid(Sigma-Aldrich) and 10% human serum (Biowest, Cat# S4190C). Activated T cells cultured with anti-CD3 and anti-CD28 in X-vivo 15 medium without cytokines served as Th0 controls. All cells were incubated at 37 °C with 5% CO_2_ for 72 h.

### Antibodies

The following antibodies were used: LPXN (Santa Cruz Biotechnology, Cat# sc-376903), IKZF4 (Santa Cruz Biotechnology, Cat# sc-292209), mouse m-IgGκ BP–HRP secondary antibody (Santa Cruz Biotechnology, Cat# sc-516102), FOXP3 (eBioscience, Cat# 14-4776-82), CTLA4 (Abcam, Cat# ab231949), IKZF3 (Abcam, Cat# ab139408), β-Actin (Sigma-Aldrich, Cat# A5441), CD45RA–FITC (BD Biosciences, Cat# 555488), CD45RO–PE (BD Biosciences, Cat# 555493), HRP-conjugated goat anti-rabbit IgG (BD Biosciences, Cat# 554021), CD160–Alexa Fluor 488 (eBioscience, Cat# 53-1609-42), FOXP3–PE (eBioscience, Cat# 12-4777-41), T-bet–BV711 (BD Pharmingen, Cat# 563320), GATA3–PE (eBioscience, Cat# 12-9966-42) and CCR6–PE (BD Pharmingen, Cat# 551773).

### Sample Preparation for Mass-Spectrometry Analyses

The iTreg and control Th0 cells were collected in LoBind tubes (Eppendorf), washed with PBS, and lysed in a buffer containing 100 mM Tris-HCl (pH 8.0) and 4% SDS. Following sonication and centrifugation at 16,000*g*, the protein-containing supernatant was collected and stored at −80 °C. For proteomic analysis, lysates were reduced with DTT and alkylated with iodoacetamide. Protein digestion was performed using the suspension-trapping (S-Trap) method as previously described (29). Briefly, DTT and iodoacetamide treated lysates were acidified with phosphoric acid, mixed with binding buffer (90% methanol, 100 mM TEAB, pH 7.1), and loaded onto S-Trap columns. After centrifugation, columns were washed with binding buffer, and proteins were digested overnight at 37 °C with MS-grade trypsin. Peptides were recovered by stepwise elution and resuspended in 2% acetonitrile/0.1% formic acid for subsequent LC–MS/MS analysis.

### MS Data Acquisition and Analysis

The resuspended peptides (800 ng) were analyzed using an EasynLC 1200 system coupled to an Orbitrap Exploris 480 mass spectrometer (Thermo Fisher Scientific). Peptides were loaded onto a pre-column (20 × 0.1 mm) and separated over a 120-min gradient on a 75 μm × 25 cm analytical column (packed in-house with 3 μm Reprosil C18; Dr Maisch GmbH). Gradient elution was performed at a flow rate of 300 nl/min, with the solvent B composition ranging from 5% to 21% in 62 min, up to 36% in 48 min, and to 100% in 5 min, with a final 5-min isocratic composition. The mobile phase consisted of water with 0.1% formic acid (A) and 80% acetonitrile with 0.1% formic acid (B). The column was maintained at 60 °C using an in-house built oven (30).

The LC-MS configuration combined a high-field asymmetric ion mobility spectrometry interface, which was operated with two compensation voltages, −40 V and −60 V. Data Independent Analysis was performed in positive-ion mode with a MS1 mass resolution of 120k, scan range of 395 to 1005 m/z, AGC target of 700,000, and cycle time of 3 s, with a maximum injection time of 50 ms. The MS/MS scan range was set to 180 to 2000 m/z, with loop control set to All. Normalized collision energy was set to 30%, Orbitrap resolution to 30k, maximum injection time of 52 ms, and AGC target to custom. A total of 30 DIA windows were applied using variable window widths optimized according to precursor density, ranging from 9 to 153 m/z. A nonoverlapping window scheme was used. Additional acquisition details are provided in the Supplementary file (Method Summary).

Raw files were converted to htrms files using the htrms converter (Biognosys), followed by directDIA database search using Spectronaut (v18, Biognosys). Protein identification was performed against the UniProtKB *Homo sapiens* reviewed database (January 2023) containing 20,435 entries). Carbamidomethylation was set as a fixed modification, while methionine oxidation and N-terminal acetylation were included as variable modifications. Precursor and fragment ion mass tolerances were approximately 5 ppm and 11 ppm, respectively. FDRs were set to 0.01. The MS raw and htrms converted files have been deposited to ProteomeXchange *via* PRIDE, project accession ID: PXD052438 (31).

For statistical analysis of the data, the protein report from Spectronaut was imported to Perseus software (1.6.2.3) (https://maxquant.net/perseus/). The data were log2 transformed and filtered to include proteins with valid values in 50% of the runs. No imputation for missing values was made. For statistical analysis a *t* test was made and the *p*-values corrected for multi hypothesis testing based on permutation.

### Sample Preparation and Analysis for Targeted Validations

The targeted SRM-MS Tier two assay was used to validate selected proteins that were differentially expressed between the iTreg and Th0 cells. Additional cultures prepared from CD4^+^ cells isolated from the cord blood of three donors were used for these measurements. As indicated in supplemental Table S8, unique peptides were chosen as SRM targets for each protein. For each analysis of the cell digests (800 ng), synthetic heavy labelled analogues of the peptide targets and retention time standards (PEPotec grade 2, produced by Thermo Fisher Scientific) were included. The samples were analyzed in triplicate in a randomized order.

An Easy-nLC 1000 liquid chromatography (Thermo Fisher Scientific) coupled to a TSQ Vantage Triple Quadrupole Mass Spectrometer (Thermo Fisher Scientific) was used for the validations. For these analyses, the above-mentioned mobile phase compositions were used at a flow rate of 300 nl/min with the following gradient; 8% to 43% B in 16 min, 43% to 100% B in 3 min, 100% B for 7 min.

Skyline software (version 21.2.0.425) (https://skyline.ms/project/home/software/Skyline/begin.view) was used to select the transitions for the assays, process the data and make the quantitative comparison between the Th0 and iTreg cells. The data were normalized using the summed GAPDH peptides as a global standard and using the Tukey’s median polish method. The raw SRM data have been deposited to the ProteomeXchange Consortium *via* the Panorama (32) with the dataset identifier PXD052690.

### Immunoblot Analysis

The cells were disrupted by sonication in RIPA buffer (Pierce, Cat# 89901) for five cycles (30 s on/30 s off) under ice-cold conditions (Bioruptor UCD-200; Diagenode), followed by centrifugation at 16,000*g* for 20 min at 4 °C. The resulting lysate was boiled in sample loading buffer and subjected to SDS-PAGE gel electrophoresis (Bio-Rad). The separated proteins were then transferred onto nitrocellulose membranes (Bio-Rad) and probed with the selected antibodies, as indicated above. The chemiluminescent signals were visualized using the Pierce ECL substrate kit (Pierce) and detected on film (Fujifilm) by autoradiography (33).

### TaqMan Quantitative Real-Time PCR

RNA was extracted from the cells using the RNeasy kit (QIAGEN, Cat# 74106). To remove DNA, an on-column DNase digestion (using RNase-Free DNase Set; QIAGEN, Cat# 79254) and an additional DNase digestion in solution (using DNase I; Invitrogen, Cat# 18068-015) were carried out.

After quantification using NanoDrop 2000, the DNase-treated RNA was reverse transcribed into cDNA using Invitrogen's SuperScript II Reverse Transcriptase kit (Thermo Fisher Scientific Cat# 18064-014), according to the manufacturer's instructions. For gene expression measurements, we used the following TaqMan Gene Expression Assays (Thermo Fisher Scientific): *LPXN* (Assay ID: Hs00183105_m1); *HIC1* (Assay ID: Hs00359611_s1); *PDCD1* (Assay ID: Hs01550088_m1); *FOXP3* (Assay ID: Hs01085834_m1); *SATB1* (Assay ID: Hs00962580_m1) (Thermo Fisher scientific). The following primers and probe synthesized by Sigma-Aldrich, were used to measure endogenous control, *EF1α* gene: forward primer 5′-CTGAACCATCCAGGCCAAAT-3′, reverse primer 5′-GCCGTGTGGCAATCCAAT-3′, probe FAM-5′-AGCGCCGGCTATGCCCCTG-3′-TAMRA (34). The TaqMan qRT-PCR runs were carried out on Applied Biosystems QuantStudio 12K Flex Real-Time PCR System and data was analyzed using QuantStudio software (https://www.thermofisher.com/fi/en/home/global/forms/life-science/quantstudio-6-7-flex-software.html?utm_source=chatgpt.com&erpType=Global_E1). The cycle threshold (Ct) value of the loading control (EF1α) was subtracted from the Ct value of the gene of interest to obtain ΔCt values. Relative mRNA expression levels were calculated using the 2^–ΔΔCt^ method, and the resulting normalized values were plotted accordingly.

### LPXN Gene Silencing

The cord blood-derived CD4^+^CD25^−^ T cells were suspended in Opti-MEM I (Invitrogen) and transfected with 300 pM *LPXN* targeting siRNAs (siRNA1: 5′-UAUUCCAACCCAGCUC-CUC-3′ or siRNA2: 5′-GGCGCAGCUCGUGUAUACUACCAAU-3′) (35), using the Nucleofector device (Lonza) and U-014 program (4 × 10^6^ cells per nucleofection). The control group received an equivalent amount of nontargeting scramble siRNA (SCR) (5′- AAUUCUCCGAACGUGUCACGU-3′) (36). Following transfection, the cells were rested in RPMI 1640 medium (Sigma-Aldrich) supplemented with penicillin/streptomycin, L-glutamine (2 mM), and 10% fetal calf serum at 37 °C for 24 h. The cells were then cultured under iTreg conditions, as described above. After 72 h of differentiation, LPXN, FOXP3, IKZF4, CTLA4, IKZF3, and β-actin levels were measured by immunoblotting in both SCR-treated and LPXN-siRNA–treated iTreg. The SCR- and LPXN-siRNA treated cells were subsequently used for the suppression assay.

### Suppression Assays

To assess the suppressive capacity of SCR- and *LPXN*-siRNA treated cells on the proliferation of effector cells (responder cells), a mixed lymphocyte reaction was performed. Responder cells (Tres) were isolated from peripheral blood buffy coat using Dynal CD4^+^ isolation (Invitrogen) and CD25 depletion (Miltenyi Biotec) kits, then stored in −150 °C freezer in freezing medium (90% FCS and 10% DMSO) to minimize variability. On day 1 of the experiment, responder cells were labeled with cell trace violet (Thermo Fisher Scientific), and 5 × 10^4^ responder cells were cultured in separate wells with SCR-treated or *LPXN*-siRNA-treated cells at ratios (iTreg: responder cells) of 1:1, 1:0.5, and 1:0.25. At the end of the 4-days assay, the division of responder cells was assessed by dye dilution. Suppression was calculated and expressed as a percentage using the following formula: % suppression = [% of dividing cells (Tres – iTreg)/% of dividing cells in Tres] × 100 (20).

### Cell Sorting

For CD160 staining, iTreg at 72 h of differentiation were washed with FACS buffer (2% fetal bovine serum, 0.01% sodium azide in PBS) and stained with AF488-conjugated CD160 antibody for 30 min at 4 °C. CD160^+^ and CD160^–^ iTreg cell sorting was performed from four replicate using a Sony SH800 Cell Sorter (Sony Biotechnology). To further characterize the CD160^+^ and CD160^–^ sorted iTreg, we conducted both suppression assays and DIA-MS analysis. The latter was performed using the EasynLC 1200 and Orbitrap Exploris 480 mass spectrometer (Thermo Fisher Scientific), as described above. The MS raw and htrms converted files have been deposited to ProteomeXchange *via* PRIDE, project accession ID: PXD052438 (31).

## Results

### Proteomic Analysis of *in Vitro* Induced Regulatory T Cells

To characterize the proteomic signatures of human iTreg cells, we performed DIA-MS-based proteomic profiling using three independent biological replicates per condition (Th0 and iTreg). In total, 8361 proteins (supplemental Table S1) and 129,989 peptides were identified and quantified across all samples. PCA and Pearson correlation demonstrated clear separation between Th0 and iTreg samples and confirmed the reproducibility of the measurements (supplemental Fig. S1, *A* and *B*). Differential expression analysis identified 935 significantly regulated proteins (log2 fold change >1, FDR <0.05), including 434 upregulated and 501 downregulated proteins in iTreg compared to Th0 cells (supplemental Table S2). These global proteomic differences were visualized using volcano plot (Fig. 1*A*) and further supported by hierarchical clustering (Fig. 1*B*), highlighting distinct protein expression patterns associated with iTreg differentiation.

**Fig. 1:**
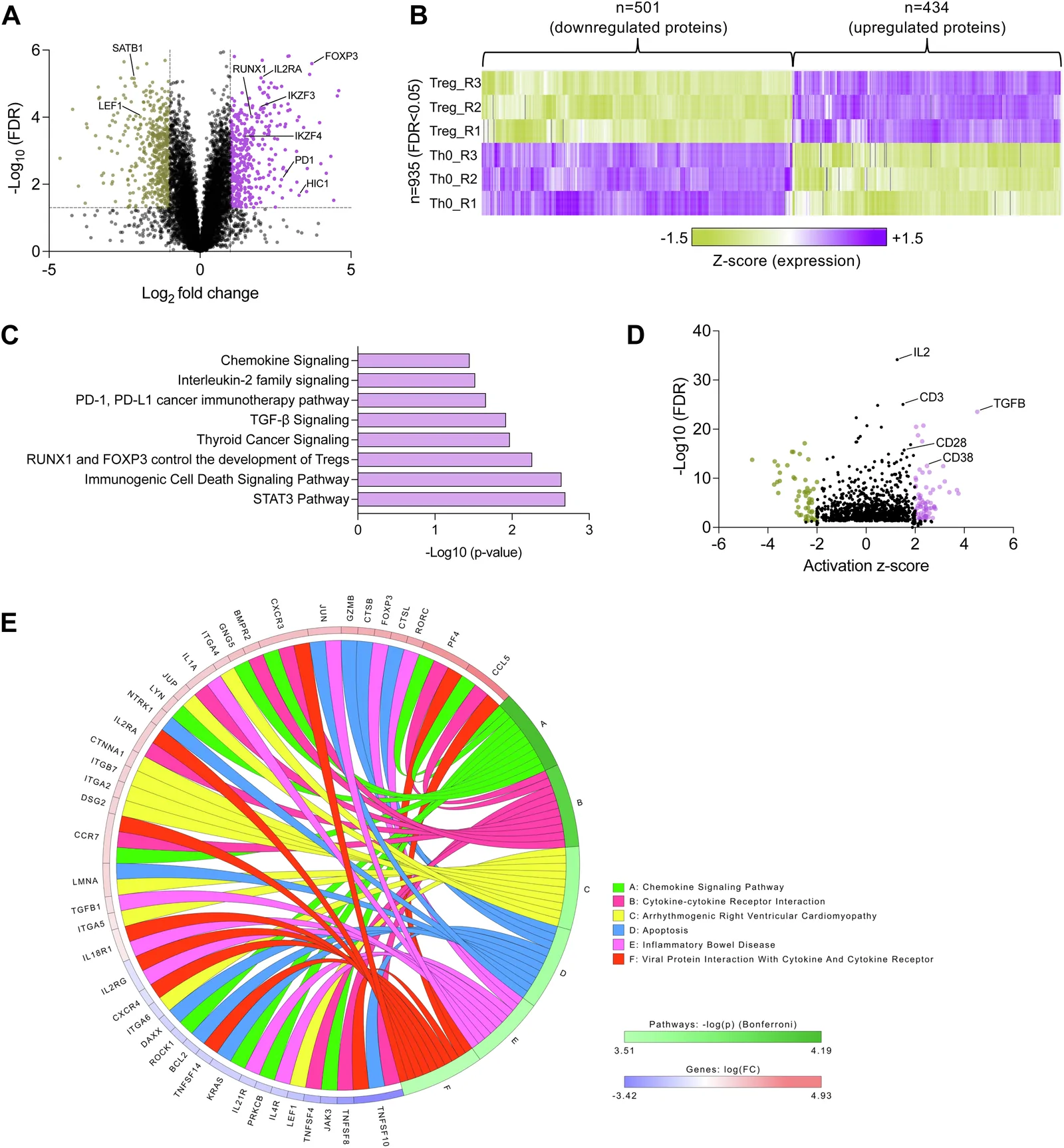
**Proteomic analysis of iTreg compared with Th0 cells.***A*, volcano plot showing proteins differentially expressed in iTreg. Proteins with FDR value ≤ 0.05 were considered significantly regulated if they presented a log2-fold change ≥1 (in *purple*) or ≤ -1 (in *green*). The volcano plot was generated using Graphpad prism (version 10). *B*, heatmap of relative protein expression values (z-scores of log_2_-transformed LFQ intensities) for the 935 differentially expressed proteins (FDR ≤0.05, log_2_FC ≥±1). The heatmap was generated using Perseus (version 1.6.2.3). *C*, the bar graph showing the top 10 enriched pathways identified using ingenuity pathway analysis. *D*, dot plot showing the top upstream regulators identified using ingenuity pathway analysis. Upstream regulators are molecules predicted to explain the observed expression changes in the dataset based on curated biological interactions, while the activation z-score predicts the activation or inhibition state of these regulators. Regulators with FDR ≤0.05 and activation z-scores ≥±2 are highlighted in *purple* and *green*. *E*, iTreg-specific pathways and biological processes identified using Advaita Bio’s iPathwayGuide analysis platform. FDR, false discovery rate.

To collate the cellular locations and functional protein classes of the differentially expressed proteins, the dataset was analyzed using QIAGEN Ingenuity Pathway Analysis (37). The results revealed that most of the differentially regulated proteins were associated with the cytoplasm, followed by the nucleus, whilst a small fraction of proteins was distributed in the plasma membrane and extracellular space (supplemental Fig. S1*C* and supplemental Table S3). The functional classes of proteins enriched in iTreg included enzymes, transcriptional regulators, and transporters, as well as cytokines and growth factors (supplemental Fig. S1*D* and supplemental Table S4). Gene ontology enrichment analysis revealed that the most enriched biological processes were associated with primary immunodeficiency signaling. As anticipated, pathways crucial to Treg function and maintenance were enriched (supplemental Table S5 and Fig. 1*C*). The top upstream regulators identified in the pathway analysis comprised of proteins, including IL2, TGFB1, CD38, CD28 and CD3 (Fig. 1*D*). The list of the top 50 identified upstream regulators and their corresponding genes is provided in supplemental Table S6. Furthermore, analysis using Advaita Bio’s iPathwayGuide (38, 39) revealed enrichment of pathways associated with chemokine signaling, apoptosis, and inflammatory bowel disease (Fig. 1*E*).

### Expression Profile of the Treg Cell Signature

To validate selected candidates, SRM-MS Tier two assays were performed in three independent biological replicates. Consistent with the expected Treg phenotype, iTreg exhibited elevated expression of multiple canonical Treg-associated proteins, including FOXP3, IL2 receptor α (IL2RA), GZMB, the zinc-finger TFs IKZF3 and IKZF4, the TNFRSF family member GITR/TNFRSF18, and the immune checkpoint receptors PD-1, CTLA-4, and LAG-3, relative to Th0 cells (Fig. 2, *A–I*). In line with our previous observations, HIC1 expression was also significantly higher in iTreg compared with Th0 cells (Fig. 2*J*) (20, 26).

**Fig. 2:**
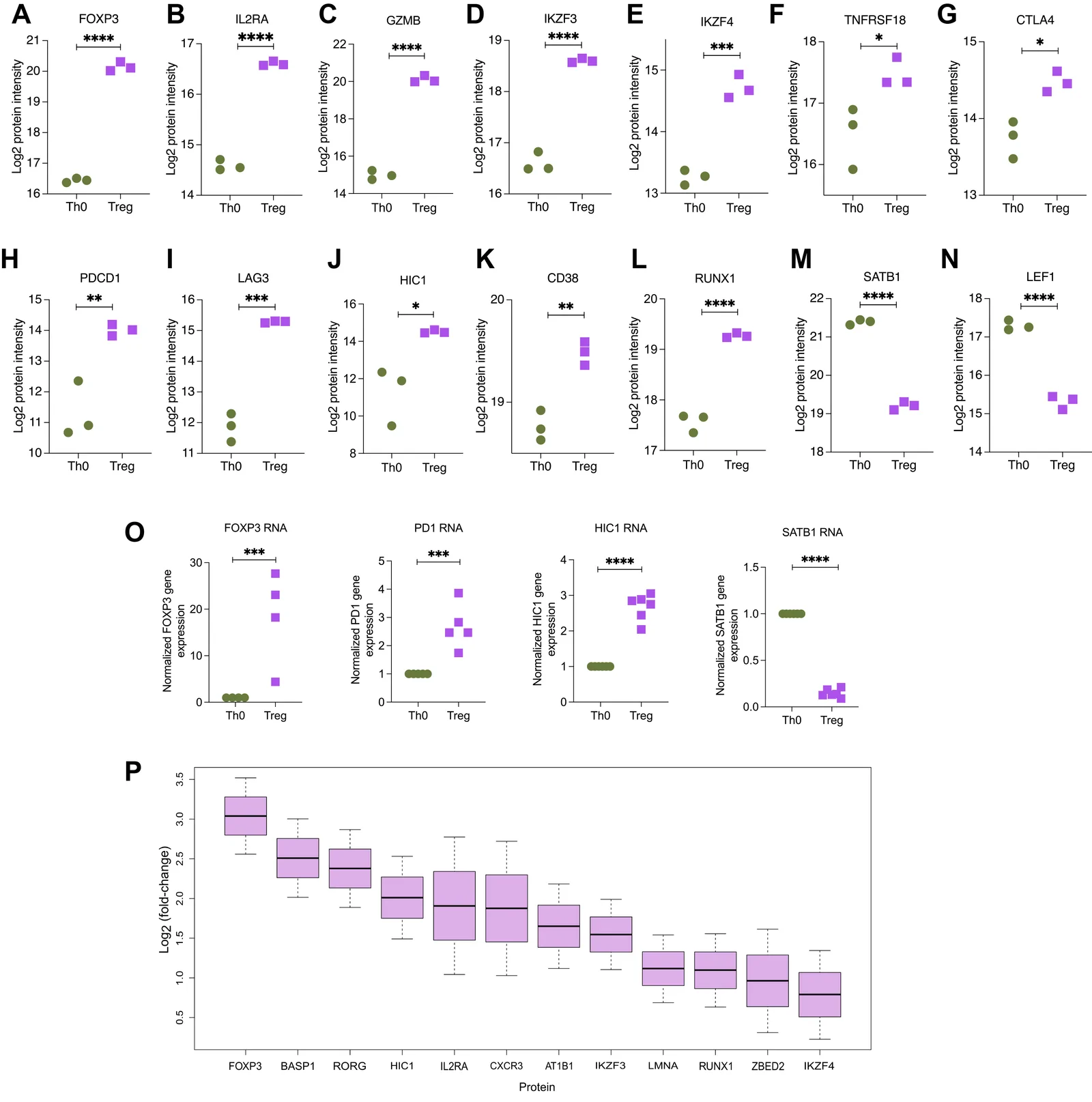
**Representation of the Treg proteomic signature.** Log_2_-transformed MS intensities of phenotypic markers (*A*) FOXP3, (*B*) IL2RA, (*C*) GZMB, (*D*) IKZF3, (*E*) IKZF4, (*F*) TNFRSF18, coinhibitory markers (*G*) CTLA4, (*H*) PDCD1, (*I*) LAG3, and other markers:, (*J*) HIC1, (*K*) CD38, (*L*) RUNX1, (*M*) SATB1, and (*N*) LEF1. *O*, qRT-PCR data is shown as Delta Ct values for the biological replicates (n > 4), normalized to EF1α, for FOXP3, PDCD1, HIC1, and SATB1 in Th0 and iTreg cells. *P*, targeted SRM-MS analysis validated the selected differentially abundant proteins, FOXP3, BASP1, RORG, HIC1, IL2RA, CXCR3, AT1B1, IKZF3, LMNA, RUNX1, ZBED2 and IKZF4. Significance was measured using two-tailed paired *t* test., ∗*p* < 0.05, ∗∗*p* < 0.005, ∗∗∗*p* < 0.0005, ∗∗∗∗*p* < 0.00005. iTreg, induced Treg.

Additionally, iTreg showed increased abundance of CD38 and RUNX1 (Fig. 2, *K–L*). CD38 is a surface glycoprotein implicated in enhancing Treg suppressive capacity, whereas RUNX1 contributes to the FOXP3 transcriptional complex that regulates the expression of key immunoregulatory genes essential for Treg function (26, 40).

Conversely, the expression of the transcriptional regulators SATB1 and LEF1—factors whose repression is important for stabilizing the Treg program (41) —was markedly reduced in iTreg compared with Th0 cells (Fig. 2, *M–N*), underscoring their roles in maintaining Treg lineage identity.

### Validation of Selected Differentially Regulated Proteins

To validate key differentially expressed Treg-associated molecules identified in our discovery dataset, TaqMan qRT-PCR and targeted proteomics approaches were used. Consistent with the proteomic findings, qRT-PCR analysis confirmed increased transcript levels of *FOXP3*, *PDCD1*, and *HIC1*, along with reduced expression of *SATB1* in iTreg compared with Th0 cells (Fig. 2*O*).

The SRM—MS analysis further corroborated differential protein abundance for a panel of selected candidates, including FOXP3, BASP1, RORG, HIC1, IL2RA, CXCR3, ATP1B1, IKZF3, LMNA, RUNX1, ZBED2, and IKZF4 (Fig. 2*P* and supplemental Table S7). These results were highly concordant with the discovery dataset. In contrast, reliable quantification of GGH and CCD25 peptides was not achieved due to weak signal intensity, preventing definitive validation of these targets.

Overall, the qRT-PCR and SRM—MS analyses supported our discovery findings and supported the robustness of the identified Treg cell protein signature.

### Novel Role of LPXN in Treg Cell Differentiation and Function

LPXN, a LIM-domain adaptor protein implicated in cytoskeletal regulation and integrin-mediated adhesion (42), has not previously been examined in the context of Treg biology. Our proteomic analysis revealed increased LPXN abundance in iTreg compared with Th0 cells (Fig. 3*A*). To confirm this finding, LPXN expression was examined over the course of differentiation using immunoblotting and qRT-PCR, for the protein and mRNA levels, respectively. These analyses confirmed a clear upregulation of LPXN during iTreg differentiation, evident at 48 h and further elevated at 72 h (Fig. 3, *B–D*).

**Fig. 3:**
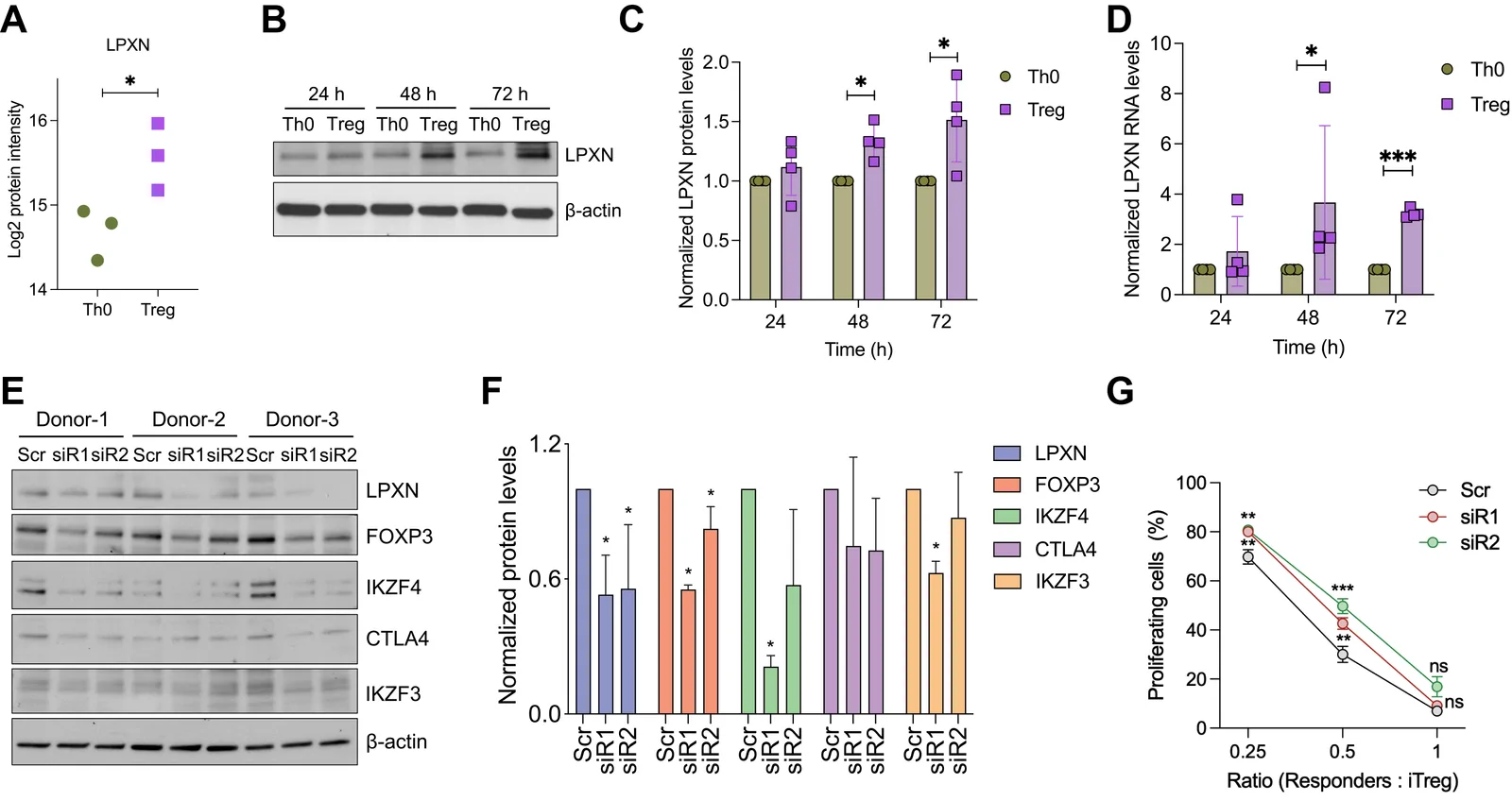
**LPXN regulates Treg cell signature proteins and function.***A*, LPXN expression in iTreg and Th0 cells in proteomics data. *B*, immunoblots of LPXN (*top*) and actin (*bottom*) in Th0 and iTreg-polarizing cells over a three-day time course. β-actin was used as a loading control. *C*, quantification of immunoblots from four replicates was performed using ImageJ. LPXN intensity values, normalized to actin, are shown as a bar graph, with each dot representing an individual replicate. *D*, qRT-PCR analysis for measurement of *LPXN* mRNA levels in Th0 and iTreg, over a three-day time-course. Delta Ct values, normalized to the control *EF1α*, are shown for four biological replicates. *E*, immunoblots of LPXN, FOXP3, IKZF4, CTLA4 and IKZF3 in control (Scr) and *LPXN* knock-down cells (siR1 and siR2) across three biological replicates. β-actin was used as a loading control. *F*, *bar plots* represent immunoblot-based quantification of LPXN, FOXP3, IKZF4, and CTLA4 in control (Scr) and *LPXN* knockdown (siR1 and siR2) iTreg. Data represents the mean ± SD of three biological replicates. *G*, the *line plot* shows the percentage of proliferating responder cells at different responder/suppressor ratios after 96 h of activation in the presence of control (Scr) or *LPXN* silenced (siR1 and siR2) iTreg. Significance was measured using two-tailed paired *t* test: ∗*p* < 0.05, ∗∗*p* < 0.005, ∗∗∗*p* < 0.0005, ‘‘ns’’ indicates not significant. iTreg, induced Treg.

To investigate the functional relevance of LPXN in the differentiation of Treg, we performed siRNA-mediated knockdown experiments. *LPXN* silencing resulted in reduced expression of key Treg-associated markers, including FOXP3, IKZF4, CTLA4, and IKZF3. (Fig. 3, *E* and *F*). Notably, *LPXN*-deficient iTreg exhibited a significant impairment in suppressive capacity relative to control siRNA-treated cells (Fig. 3*G*). Together, these findings identify LPXN as a previously unrecognized regulator of human iTreg differentiation and suppressive function, supporting a role for this adaptor protein in stabilizing the Treg transcriptional and functional program.

### CD160 Marks a Distinct Subpopulation of iTreg

CD160 is a cell-surface receptor expressed on several immune cell types including NK cells, NK T cells and activated T cells (42, 43). Proteomic analysis revealed higher CD160 expression in iTreg compared with Th0 cells (Fig. 4*A*) and demonstrated progressive upregulation from 24 to 72 h of differentiation (Fig. 4*B*). To determine CD160 expression across T helper cell lineages, naïve CD4^+^ T cells were polarized into Th1, Th2, Th17, and iTreg subsets, with Th0 used as the TCR—activated control. CD160 expression was restricted to iTreg, with no detectable expression in other differentiated CD4^+^ lineages (Fig. 4*C*). To identify factor(s) that induce CD160 expression, T cells were stimulated with combinations of Th-polarizing cytokines. All conditions containing TGF-β upregulated CD160 at both the mRNA and protein levels (Fig. 4, *D* and *E*), indicating that CD160 expression is upregulated by TGF-β. To further characterize CD160^+^ iTreg, we sorted CD160^+^FOXP3^+^ and CD160^-^FOXP3^+^ populations and compared their suppressive function. Both subsets exhibited comparable suppressive activity (Fig. 4*F*). However, proteomic profiling revealed substantial proteomic differences between them. Distinct patterns of protein expression were observed between the two populations, as reflected in the PCA analysis (supplemental Fig. S3*A*). In total, 8241 proteins were quantified, and 750 were differentially abundant between the two populations (FDR <0.05, log_2_FC >0.58), including 446 upregulated and 304 downregulated proteins in CD160^+^ iTreg (supplemental Table S9 and supplemental Fig. S3*B*). As expected, CD160 was among the most enriched proteins in CD160^+^ iTreg (Fig. 4*G*). CD160 expression correlated positively with IRF2 and NR4A2, which have been reported to act as a negative regulator of proinflammatory signaling and as a promoter of FOXP3 induction and Treg differentiation, respectively (18, 43). CD160^+^ iTreg were also enriched for programmed cell death regulators (PDCD10, PDCD6) and multiple zinc-finger proteins (*e*.*g*., ZNF700, ZNF703, ZNF107, ZNF9, ZNF365, ZNF551). In addition, key immune regulatory factors such as RUNX2, FOSL2, and NFKBIL1 were elevated (44, 45, 46), whereas STAT4, a known inhibitor of Treg suppressive function (23), was reduced in CD160^+^ iTreg. Conversely, the CD160^+^ iTreg showed lower abundance of several immune regulatory factors, including FOXO1, IKZF5, TNFRSF18, BATF, and NFYA. Together, these findings demonstrate that CD160 marks a distinct iTreg subpopulation.

**Fig. 4:**
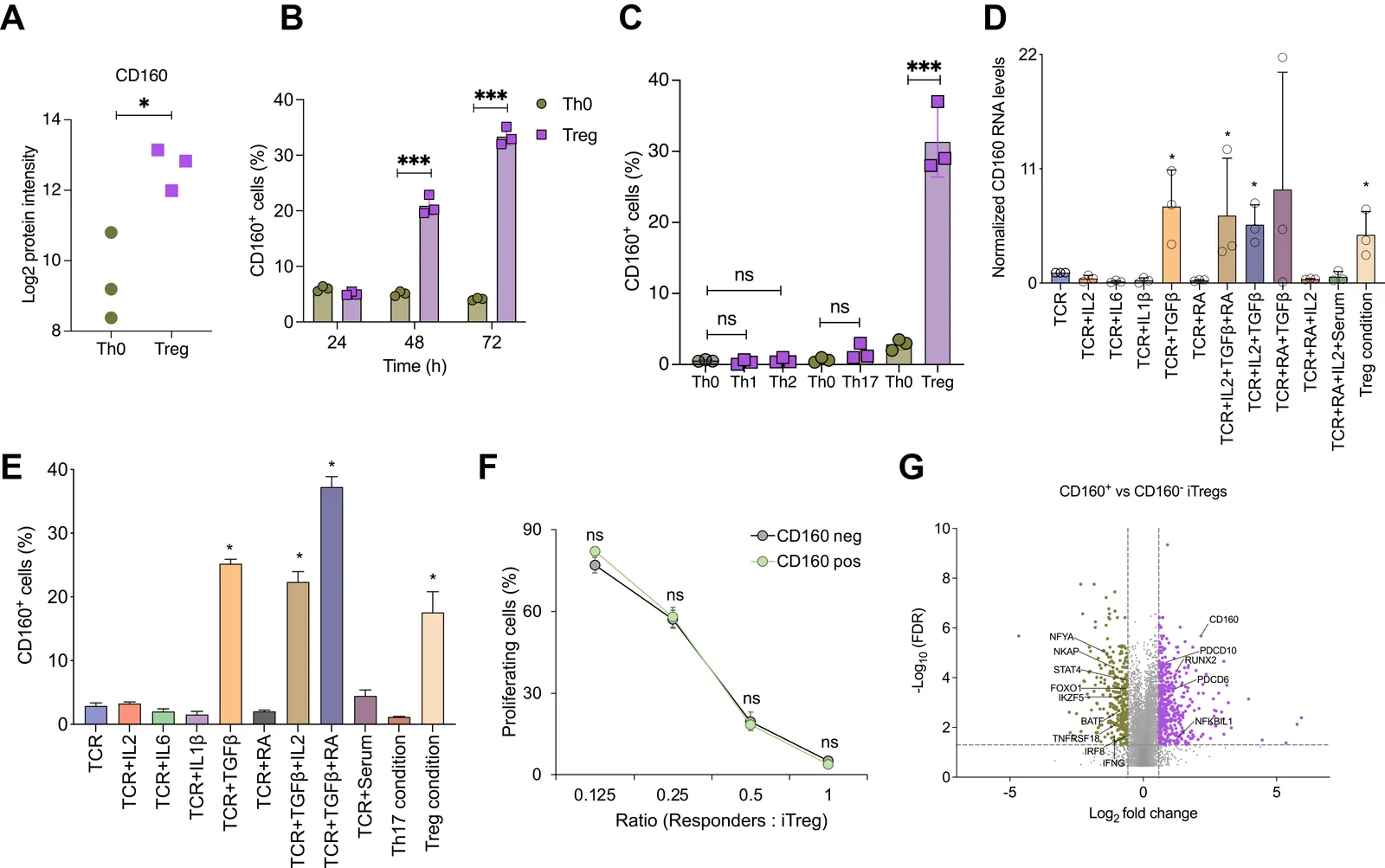
**CD160 is differentially expressed in a subpopulation of iTreg cells.***A*, CD160 expression in iTreg and Th0 cells in proteomics data after imputation. *B*, flow cytometry analyses show % of CD160^+^ cells in Th0 and iTreg-polarizing cells, over a three-day time course. *C*, CD160 expression is restricted to iTreg, with no detectable signal in *in vitro* differentiated Th1, Th2, or Th17 cells as assessed by flow cytometry. TGF-β induced CD160 expression across the cytokine combinations tested, as shown by (*D*) qRT-PCR and (*E*) flow cytometry. *F*, the percentage of proliferating responder cells at different responder/suppressor ratios are shown after 96 h of activation in the presence of CD160^+^ or CD160^−^ iTreg. No significant difference in suppressive ability was observed between CD160^+^ and CD160^−^ iTreg. *G*, DIA-MS proteomics analysis was performed on sorted CD160^+^ and CD160^−^ iTreg for four biological replicates. The *volcano plot* highlights the proteins that are differentially expressed between CD160^+^ and CD160^−^ iTreg subpopulations at 72 h of polarization with FDR ≤0.05 and log_2_FC ≥ 0.58. Upregulated proteins are in purple, and downregulated proteins are in *green*. Significance was measured using two-tailed paired *t* test. ∗*p* < 0.05, ∗∗∗*p* < 0.0005, ∗∗∗∗*p* < 0.00005, ‘‘ns’’ indicates not significant. iTreg, induced Treg.

## Discussion

In this study, DIA-MS–based proteomic profiling provided broad coverage of the protein landscape distinguishing human iTreg from Th0 cells. Validation using TaqMan qRT-PCR and targeted proteomics confirmed key proteins of the iTreg signature, including increased expression of FOXP3, IL2RA, CTLA4, PDCD1, and HIC1, together with reduced SATB1, reflecting the well-established transcriptional programs that support Treg differentiation and suppressive activity (20, 22). Canonical pathways relevant to Treg biology—including IL-2 signaling, PD-1/PD-L1 immunoregulation, TGF-β signaling, and RUNX1–FOXP3 regulatory network—were enriched in iTreg, underscoring the robustness of the dataset.

Our study presents deep proteomic profiling of human iTregs induced under a defined protocol that has been demonstrated in our earlier studies. Comparison of this data with the previous iTreg proteomics data by Schmidt *et al*. (22) revealed approximately 200 commonly differentially regulated proteins, including key regulators such as HIC1, ICOS, IKZF3 and FOXP3. Further details of the comparison are provided in supplemental Table S10.

An important consideration is how the identity of iTreg relates to tTreg. Mensink *et al*. (2022) compared iTreg, tTreg, and Tconv using a similar DIA-based approach, identifying ∼4500 proteins, including 475 differentially regulated proteins across the cell types. While the direct comparison of our findings is limited by differences in reference conditions, there were 65 common proteins between the differentially expressed proteins. This overlap suggests that while iTreg exhibit a distinct proteomic identity, many of the differences identified in our study may also be relevant to tTreg populations. Further details of this comparison are provided in supplemental Table S11. The list of the proteins common in the three studies is shown in supplemental Table S12.

Data from this study revealed elevated LPXN expression in iTreg and demonstrated that LPXN contributes to maintaining the expression of key Treg markers and suppressive activity. These findings highlight LPXN’s role in Treg development and function, suggesting its potential as a therapeutic target for modulating Treg-mediated immune response. Notably, cross-reference to the Human Protein Atlas (47) revealed that the highest expression of *LPXN* in immune cells is observed in Treg (supplemental Fig. S4). A direct comparison with the data from the study of Mensink revealed that LPXN was detected at similar levels in the tTreg, iTreg and Tconv cells, supporting the reproducibility of LPXN detection across datasets. Consistent with our findings, LPXN was significantly increased upon iTreg induction in the dataset reported by Schmidt *et al*.

LPXN is known to regulate T cell activation and migration by colocalizing with LFA-1. This interaction might be essential for Treg cell function, as it regulates the formation of immunological synapses and migration (48, 49, 50). The interaction of LPXN with LFA-1, and other proteins, such as β-catenin (51), which is a key modulator of the Wnt signaling pathway that is central to Treg cell development and function (11), suggests that LPXN may regulate Treg suppression by interacting with key Treg proteins. In addition, Leupaxin plays an inhibitory role in B cell function by inhibiting the JNK and p38 MAPK signaling pathways downstream BCR signaling and is dispensable for T-dependent and T-independent B cell immune responses in mice (49, 52). The signaling pathways downstream of LPXN represent an important area for future investigation.

This study identifies CD160 as a marker associated with a distinct iTreg subpopulation. CD160 is expressed by several lymphocyte subsets, including T cells, and binds weakly to MHC class I molecules but with higher affinity to HVEM/TNFRSF14 (53). In CD4+ T cells, CD160 was shown to be upregulated following TCR/CD28 stimulation, and engagement of CD160 by HVEM inhibited CD4+ T-cell activation, including proliferation and cytokine production (54). In CD8+ T cells, CD160 has a more context-dependent role: it can function as either a co-activating or co-inhibitory receptor. CD160 has been associated with inhibitory/exhaustion-related programs during chronic stimulation, but also with costimulatory effector functions (55, 56, 57).

CD160 expression was restricted to iTreg among the CD4^+^ T cell lineages examined and was induced by TGF-β. Although CD160^+^ and CD160^−^ iTreg exhibited comparable suppressive activity, proteomic analyses revealed distinct molecular differences between the two populations. Specifically, CD160^+^ iTreg were enriched for regulators of transcription and immune modulation—including IRF2, NR4A2, RUNX2, FOSL2, and NFKBIL1. These findings suggest that CD160 defines a distinct iTreg subset with potential context-dependent roles in immune regulation, which could be the subject of further investigations. In an earlier study, we identified a CD103+ iTreg subpopulation with enhanced suppressive capacity (58). Interestingly, although CD103 abundance did not differ between CD160+ and CD160−iTreg populations, it is noteworthy that ITGB7 was among the proteins enriched in the overall iTreg proteome. Given that heterodimerization with ITGB7 is required for stable surface expression of CD103 (59), this observation may further support the relevance of integrin-mediated processes in iTreg biology.

Beyond canonical Treg markers, the proteomic analysis revealed differential expression of additional regulators critical to Treg development and function. Members of the NR4A family (NR4A1, NR4A2, and NR4A3), TFs involved in T cell activation, exhaustion, and tolerance (18, 60, 61), were consistently elevated in iTreg. These TFs play a well-established role in promoting FOXP3 expression, reinforcing Treg lineage commitment, and suppressing effector programs (62), supporting their key role to iTreg identity. We also observed high expression of ITGB7 in iTreg, a gene encoding the β7 integrin subunit. ITGB7 forms heterodimers with α4 or αE integrins to mediate Treg trafficking to gut-associated lymphoid tissues, where it supports localized immunosuppressive function and the maintenance of immune tolerance (63, 64). Additionally, our data show increased expression of the vitamin D receptor (*VDR*) in iTreg. Upon activation, VDR signaling enhances *FOXP3* expression and promotes the differentiation and suppressive capacity of Tregs. Impaired VDR activity has been associated with reduced Treg function, underscoring its importance in sustaining immune tolerance (65).

Together, these data define a comprehensive proteomic signature of human iTreg, expanding the molecular understanding of their differentiation and function. The identification of LPXN as a regulator of Treg stability and suppressive activity, along with the delineation of a CD160^+^ iTreg subpopulation, provides new insights into Treg heterogeneity. These findings offer a valuable resource for mechanistic studies and provide a basis for future therapeutic strategies aimed at modulating Treg responses in immune-mediated disorders.

## Data Availability

All raw mass spectrometry data and search results have been deposited to the ProteomeXchange Consortium *via* the Pride partner repository with the data set identifier PXD052438. The raw SRM data have been deposited to the ProteomeXchange Consortium *via* the Panorama with the dataset identifier PXD052690.

## Supplemental Data

This article contains supplemental data (20, 66).

## Conflict of Interest

The authors declare no competing interests.
