# Supplementary material for: Proteomic Insights Into Human Induced Regulatory T Cells: The Role of LPXN and CD160 in Immune Suppression
Source: Mol Cell Proteomics. 2026 Aug 20;25(10):101644. doi: 10.1016/j.mcpro.2026.101644 (PMC13627119; doi:10.1016/j.mcpro.2026.101644)
Supplement: Supplementary file 1 [file mmc1.pdf]

Proteomic insights into human induced regulatory T cells: the role of LPXN and CD160 in immune suppression

Supplementary methods

Th1 and Th2 cell polarization: Human mononuclear cells (MNCs) were isolated from umbilical cord blood by density-gradient centrifugation using Ficoll-Paque Plus (Cytiva, Cat# 17144003). $CD4^+$ T cells were purified from MNCs via magnetic sorting with $CD4^+$ Dynal positive selection beads (Invitrogen, Cat# 11331D), as previously described (1). Prior to activation, naïve $CD4^+$ T cells from multiple donors were identified by their $CD45RA^+CD45RO^-$ phenotype using flow cytometry.
$CD4^+$ T cells were activated with plate-bound anti-CD3 (500 ng/mL; Beckman Coulter, Cat# IM1304) and soluble anti-CD28 (500 ng/mL; Beckman Coulter, Cat# IM1376) in Yessel medium (Iscove's Modified Dulbecco's Medium, cat # 31980-048, Invitrogen) (2). Th1 polarization was induced with IL-12 (2.5 ng/mL), and Th2 polarization with IL-4 (10 ng/mL) (both from R&D Systems). For differentiation controls, activated Th0 cells were stimulated with anti-CD3 and anti-CD28 under identical conditions but without polarizing cytokines.

Th17 cell polarization: Human mononuclear cells (MNCs) were isolated from umbilical cord blood by density-gradient centrifugation using Ficoll-Paque Plus (Cytiva, Cat# 17144003). $CD4^+$ T cells were purified from MNCs by magnetic sorting with $CD4^+$ Dynal positive selection beads (Invitrogen, Cat# 11331D), as previously described (1). Naïve $CD4^+$ T cells from multiple donors were confirmed by flow cytometry based on their $CD45RA^+CD45RO^-$ phenotype.

CD4⁺ T cells were activated with plate-bound anti-CD3 (3.75 µg/well; Beckman Coulter, Cat# IM1304) and soluble anti-CD28 (1 µg/mL; Beckman Coulter, Cat# IM1376) in X-VIVO 20 serum-free medium (Lonza). Th17 polarization was induced with a cytokine cocktail consisting of IL-6 (20 ng/mL), IL-1β (10 ng/mL), and TGF-β (10 ng/mL), together with neutralizing antibodies against IFN-γ (1 µg/mL) and IL-4 (1 µg/mL) to block Th1 and Th2 differentiation, respectively.

For differentiation controls, Th0 cells were generated by stimulating naïve $CD4^+$ T cells with the same plate-bound anti-CD3 (3.75 µg/mL) and soluble anti-CD28 (1 µg/mL) antibodies in X-VIVO 20 medium without polarizing cytokines. X-VIVO 20 medium was supplemented with L-glutamine (2 mM; Sigma-Aldrich) and antibiotics (50 U/mL penicillin and 50 µg/mL streptomycin; Sigma-Aldrich). All cultures were maintained at 37°C in a humidified atmosphere containing 5% CO_2.

References

1. Ubaid Ullah, Andrabi, S. B. A., Tripathi, S. K., Dirasantha, O., Kanduri, K., Rautio, S., Gross, C. C., Lehtimäki, S., Bala, K., Tuomisto, J., Bhatia, U., Chakroborty, D., Elo, L. L., Lähdesmäki, H., Wiendl, H., Rasool, O., and Lahesmaa, R. (2018) Transcriptional Repressor HIC1 Contributes to Suppressive Function of Human Induced Regulatory T Cells. *Cell Rep.* 22, 2094–2106
2. Yssel, H., De Vries, J. E., Koken, M., Van Blitterswijk, W., and Spits, H. (1984) Serum-free medium for generation and propagation of functional human cytotoxic and helper T cell clones. *J. Immunol. Methods* 72, 219–227
