# Supplementary material for: Proteomic Insights Into Human Induced Regulatory T Cells: The Role of LPXN and CD160 in Immune Suppression
Source: Mol Cell Proteomics. 2026 Aug 20;25(10):101644. doi: 10.1016/j.mcpro.2026.101644 (PMC13627119; doi:10.1016/j.mcpro.2026.101644)
Supplement: Supplementary file 2 [file mmc3.pdf]

Proteomic insights into human induced regulatory T cells: the role of LPXN and CD160 in immune suppression

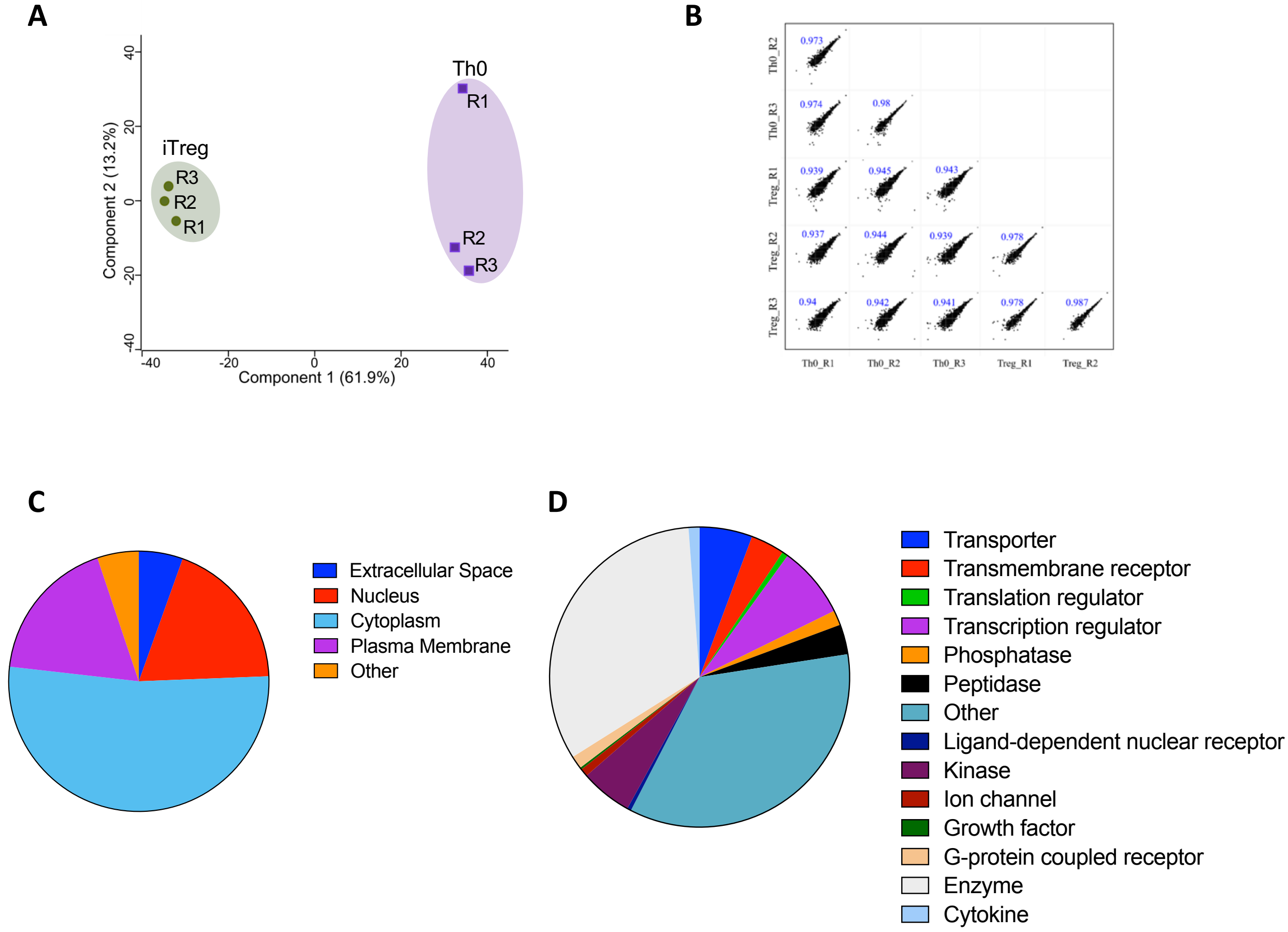

Figure S1**: **Proteomic profiles of iTreg compared to TCR-activated Th0 cells.

***(A)** PCA plot of iTreg and Th0 proteomes (green dots: iTregs; purple squares: Th0; n = 3 donors). **(B)** Multi-scatter plot showing Pearson correlation coefficients between Th0 and iTreg proteomes. The multi-scatter plot and PCA plot were generated using Perseus (version 1.6.2.3). **(C–D)** Ingenuity Pathway Analysis of differentially expressed proteins. Pie charts show the distribution of proteins by **(C)** cellular localization and **(D)** functional protein classes.*

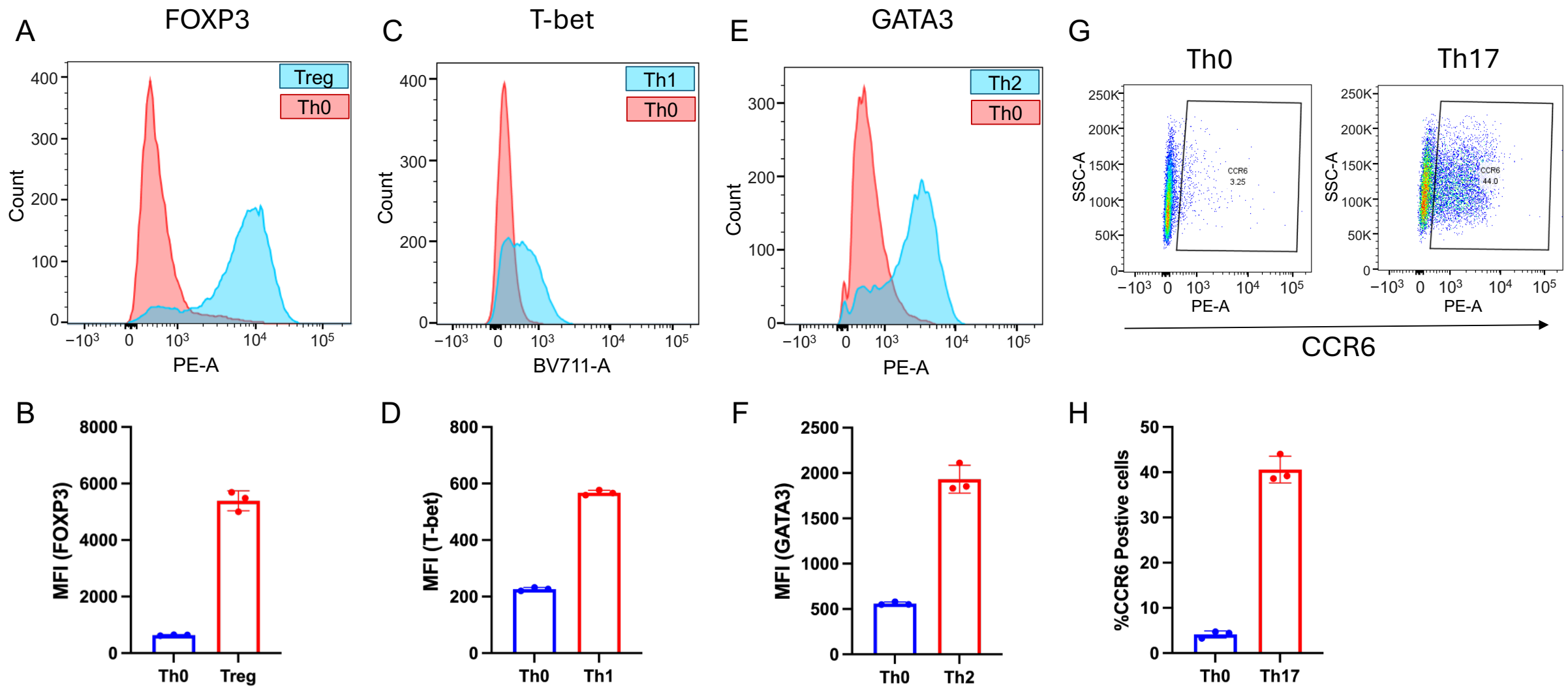

Figure S2. In vitro–induced Tregs, Th1, Th2, and Th17 cells express FOXP3, T-bet, GATA3, and CCR6, respectively.

(A–B) *Intracellular FOXP3 staining in Th0 and iTregs, showing mean fluorescence intensity (MFI).* ***(C–D)*** *Intracellular T-bet staining in Th0 and Th1 cells, showing MFI.* ***(E–F)*** *Intracellular GATA3 staining in Th0 and Th2 cells, showing MFI.* ***(G–H)*** *Percentage of CCR6^{+} cells in Th0 and Th17 cells at 72 h, assessed by flow cytometry across three biological replicates.*

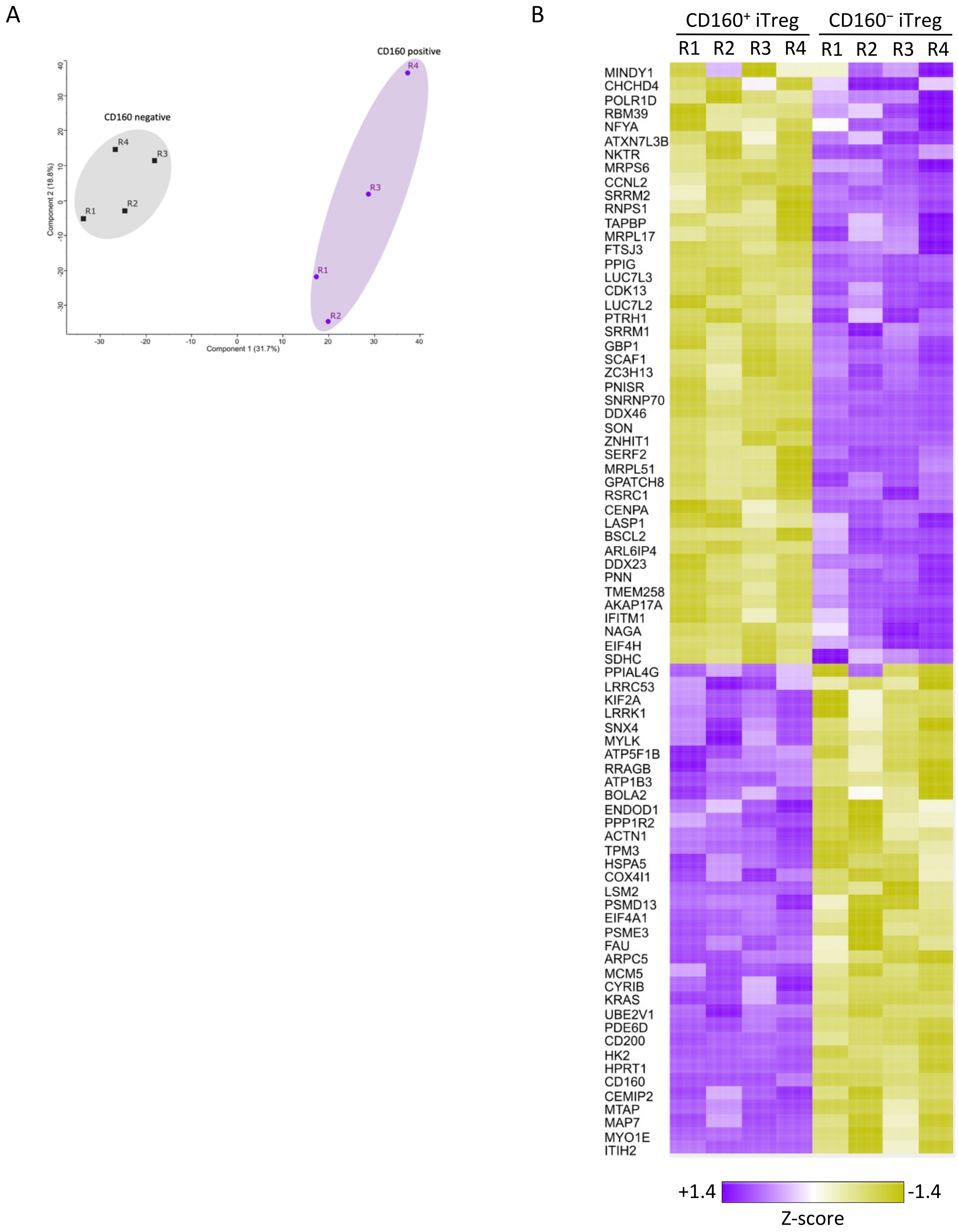

Figure S3: Proteomic profiles of $CD160^+$ iTreg compared to $CD160^-$ iTreg. (A) *Principal component analysis (PCA) showing distinct proteomic profiles of $CD160^+$ iTregs compared with $CD160^-$ iTregs.* ***(B)*** *Z-score heatmap of the top 80 significantly differentially expressed proteins (FDR < 0.05, log_2FC > 0.58) across four biological replicates.*

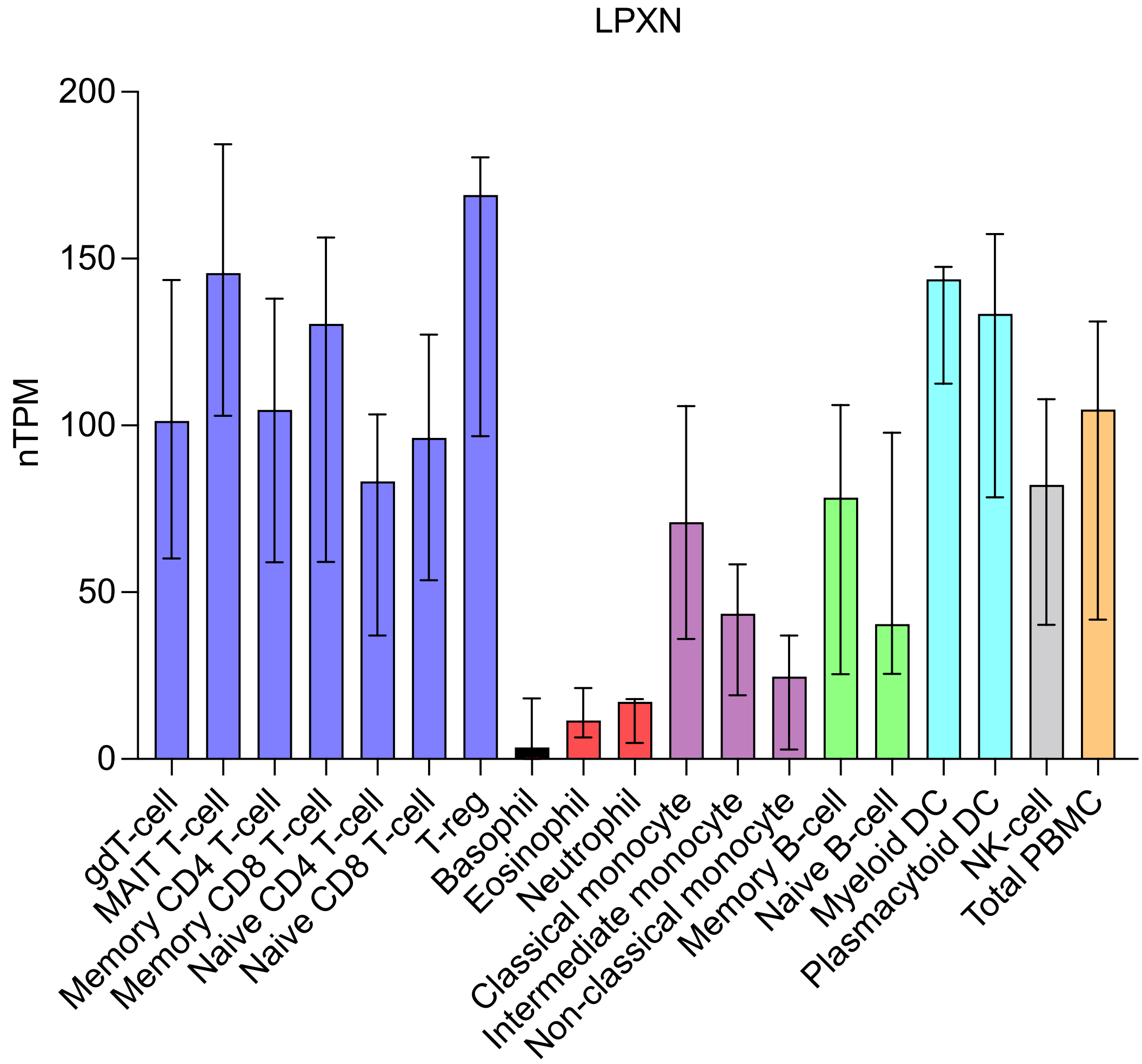

Figure S4: Expression of LPXN transcripts across immune cell types.
Normalized transcript expression values (nTPM) for LPXN were obtained from the Human Protein Atlas, and median values were plotted as a bar graph (Error bar represents maximum and minimum nTPM values). Treg cells showed the highest LPXN expression. Blue bars represent T cells; red bars, granulocytes; purple bars, monocytes; green bars, B cells; cyan bars, dendritic cells; gray bars, NK cells; and orange bars, total PBMCs.
