# Supplementary material for: Proteomic Insights Into Human Induced Regulatory T Cells: The Role of LPXN and CD160 in Immune Suppression
Source: Mol Cell Proteomics. 2026 Aug 20;25(10):101644. doi: 10.1016/j.mcpro.2026.101644 (PMC13627119; doi:10.1016/j.mcpro.2026.101644)
Supplement: Supplementary file 3 [file mmc4.pdf]

Method Summary

Method Settings

Application Mode: **Peptide**
Method Duration (min): **120**

Global Parameters

Ion Source

Ion Source Type: **NSI**
Spray Voltage: **Static**
Positive Ion (V): **2200**
Negative Ion (V): **600**
Ion Transfer Tube Temp (°C): **280**
Use Ion Source Settings from Tune: **False**
FAIMS Mode: **Standard Resolution**
Total Carrier Gas Flow: **Static**
Total Carrier Gas Flow (L/min): **3.7**

MS Global Settings

Infusion Mode: **Liquid Chromatography**
Expected LC Peak Width (s): **30**
Advanced Peak Determination: **False**
Default Charge State: **2**
Enable Xcalibur AcquireX Ab method modifications: **False**
Internal Mass Calibration: **Off**

Experiment #1 [MS]

Start Time (min): **0**
End Time (min): **120**

Master Scan:

Full Scan

Orbitrap Resolution: **120000**
Scan Range (m/z): **395-1005**
FAIMS Voltages: **On**
FAIMS CV (V): **-40**
RF Lens (%): **35**
AGC Target: **Custom**
Normalized AGC Target (%): **300**
Maximum Injection Time Mode: **Custom**
Maximum Injection Time (ms): **50**
Microscans: **1**
Data Type: **Profile**
Polarity: **Positive**
Source Fragmentation: **Disabled**
Scan Description:

Experiment #2 [tMS2]

Start Time (min): **0**
End Time (min): **120**

Master Scan:

tMS²

Multiplex Ions: **False**
Isolation Offset: **Off**
Collision Energy Type: **Normalized**
Orbitrap Resolution: **30000**
TurboTMT: **Off**
Scan Range Mode: **Define m/z Range**
Scan Range (m/z): **180-2000**
FAIMS Voltages: **On**
FAIMS CV (V): **-40**
RF Lens (%): **35**
AGC Target: **Custom**
Normalized AGC Target (%): **2000**
Maximum Injection Time Mode: **Custom**
Maximum Injection Time (ms): **52**
Microscans: **1**
Data Type: **Centroid**
Polarity: **Positive**
Source Fragmentation: **Disabled**
Loop Control: **All**
Dynamic RT: **Off**
Time Mode: **Unscheduled**
Scan Description:

Mass List Table

Mass List Table

Compound	Formula	Adduct	m/z	z	Isolation Window (m/z)	HCD Collision Energies (%)
		(no adduct)	408	2	16	30
		(no adduct)	422	2	12	30
		(no adduct)	433.5	2	11	30
		(no adduct)	444.5	2	11	30
		(no adduct)	455	2	10	30
		(no adduct)	464.5	2	9	30
		(no adduct)	474	2	10	30
		(no adduct)	483.5	2	9	30
		(no adduct)	492.5	2	9	30
		(no adduct)	502	2	10	30
		(no adduct)	511.5	2	9	30
		(no adduct)	521	2	10	30
		(no adduct)	531	2	10	30
		(no adduct)	541	2	10	30
		(no adduct)	551	2	10	30
		(no adduct)	561.5	2	11	30
		(no adduct)	572.5	2	11	30
		(no adduct)	584	2	12	30
		(no adduct)	596	2	12	30
		(no adduct)	609	2	14	30
		(no adduct)	623	2	14	30
		(no adduct)	638	2	16	30
		(no adduct)	654.5	2	17	30
		(no adduct)	672	2	18	30
		(no adduct)	691	2	20	30
		(no adduct)	712.5	2	23	30
		(no adduct)	738	2	28	30
		(no adduct)	771	2	38	30
		(no adduct)	818.5	2	57	30

(no adduct)	923.5	2	153	30

Experiment #3 [MS]

Start Time (min): **0**
End Time (min): **120**

Master Scan:

Full Scan

Orbitrap Resolution: **120000**
Scan Range (m/z): **395-1005**
FAIMS Voltages: **On**
FAIMS CV (V): **-60**
RF Lens (%): **35**
AGC Target: **Custom**
Normalized AGC Target (%): **300**
Maximum Injection Time Mode: **Custom**
Maximum Injection Time (ms): **50**
Microscans: **1**
Data Type: **Profile**
Polarity: **Positive**
Source Fragmentation: **Disabled**
Scan Description:

Experiment #4 [tMS2]

Start Time (min): **0**
End Time (min): **120**

Master Scan:

tMS^2

Multiplex Ions: **False**
Isolation Offset: **Off**
Collision Energy Type: **Normalized**
Orbitrap Resolution: **30000**
TurboTMT: **Off**
Scan Range Mode: **Define m/z Range**
Scan Range (m/z): **180-2000**
FAIMS Voltages: **On**
FAIMS CV (V): **-60**
RF Lens (%): **35**
AGC Target: **Custom**
Normalized AGC Target (%): **2000**
Maximum Injection Time Mode: **Custom**
Maximum Injection Time (ms): **52**
Microscans: **1**
Data Type: **Centroid**
Polarity: **Positive**
Source Fragmentation: **Disabled**
Loop Control: **All**
Dynamic RT: **Off**
Time Mode: **Unscheduled**
Scan Description:

Mass List Table

Mass List Table

Compound	Formula	Adduct	m/z	z	Isolation Window (m/z)	HCD Collision Energies (%)
		(no adduct)	408	2	16	30
		(no adduct)	422	2	12	30
		(no adduct)	433.5	2	11	30
		(no adduct)	444.5	2	11	30
		(no adduct)	455	2	10	30
		(no adduct)	464.5	2	9	30
		(no adduct)	474	2	10	30
		(no adduct)	483.5	2	9	30
		(no adduct)	492.5	2	9	30
		(no adduct)	502	2	10	30
		(no adduct)	511.5	2	9	30
		(no adduct)	521	2	10	30
		(no adduct)	531	2	10	30
		(no adduct)	541	2	10	30
		(no adduct)	551	2	10	30
		(no adduct)	561.5	2	11	30
		(no adduct)	572.5	2	11	30
		(no adduct)	584	2	12	30
		(no adduct)	596	2	12	30
		(no adduct)	609	2	14	30
		(no adduct)	623	2	14	30
		(no adduct)	638	2	16	30
		(no adduct)	654.5	2	17	30
		(no adduct)	672	2	18	30
		(no adduct)	691	2	20	30
		(no adduct)	712.5	2	23	30
		(no adduct)	738	2	28	30
		(no adduct)	771	2	38	30
		(no adduct)	818.5	2	57	30
		(no adduct)	923.5	2	153	30
